# Aging of Non-Visual Spectral Sensitivity to Light in Humans: Compensatory Mechanisms?

**DOI:** 10.1371/journal.pone.0085837

**Published:** 2014-01-23

**Authors:** Raymond P. Najjar, Christophe Chiquet, Petteri Teikari, Pierre-Loïc Cornut, Bruno Claustrat, Philippe Denis, Howard M. Cooper, Claude Gronfier

**Affiliations:** 1 Department of Chronobiology, Inserm U846, Stem Cell and Brain Research Institute, Bron, France; 2 University of Lyon, Claude Bernard Lyon 1, Villeurbanne, France; 3 University Joseph Fourier Grenoble 1, Grenoble, France; 4 Department of Ophthalmology, CHU Grenoble, Grenoble, France; 5 Department of Ophthalmology, CHU de Lyon Hôpital Edouard Herriot, Lyon, France; 6 Center of Biology, Hormone Laboratory, Bron, France; 7 Department of Ophtalmology, Hôpital de la Croix-Rousse, Lyon, France; McGill University, Canada

## Abstract

The deterioration of sleep in the older population is a prevalent feature that contributes to a decrease in quality of life. Inappropriate entrainment of the circadian clock by light is considered to contribute to the alteration of sleep structure and circadian rhythms in the elderly. The present study investigates the effects of aging on non-visual spectral sensitivity to light and tests the hypothesis that circadian disturbances are related to a decreased light transmittance. In a within-subject design, eight aged and five young subjects were exposed at night to 60 minute monochromatic light stimulations at 9 different wavelengths (420–620 nm). Individual sensitivity spectra were derived from measures of melatonin suppression. Lens density was assessed using a validated psychophysical technique. Although lens transmittance was decreased for short wavelength light in the older participants, melatonin suppression was not reduced. Peak of non-visual sensitivity was, however, shifted to longer wavelengths in the aged participants (494 nm) compared to young (484 nm). Our results indicate that increased lens filtering does not necessarily lead to a decreased non-visual sensitivity to light. The lack of age-related decrease in non-visual sensitivity to light may involve as yet undefined adaptive mechanisms.

## Introduction

The endogenous circadian clock, located in the hypothalamic suprachiasmatic nuclei (SCN), regulates sleep, hormonal rhythms, temperature, and a wide range of physiological functions in mammals including humans. Light is the strongest synchronizer of the clock in both human and non-human mammals [1]. Appropriate light exposure is necessary for entrainment of this timing system to the 24-hour light-dark cycle, and consequently, for appropriate timing of physiological functions. Inappropriate entrainment is associated with temperature, cardiovascular, immunological, sleep, vigilance, memory, and neurocognitive alterations [2]–[5]. Circadian photo-entrainment is mediated by intrinsically photoreceptive melanopsin expressing ganglion cells in the retina (ipRGCs) that receive input from conventional visual photoreceptors (rods, cones) and convey photic information to non-visual brain structures such as the SCN [6]–[8]. The effect of light on the circadian system is dependent on timing [9], intensity [10], [11], duration [12], wavelength (λ) [7], [13]–[15] and temporal pattern [3], [16], [17] of the light stimulus. Peak sensitivity of non-visual responses is in the blue range of the light spectrum (∼460–480 nm) in young adults [13], [15].

Aging is often associated with sleep and circadian disturbances [18]–[20]. In addition, phase and amplitude of several circadian functions have been found to be altered with age [20], [21]. These changes could be due, in part, to age-related alterations of the circadian system [22], [23], either within the eye and the retina (synchronizing input), or at the level of the central clock in the SCN [24], [25] or at output levels (brain structures involved in sleep regulation and rhythmic functions such as the ventrolateral preoptic nucleus (VLPO) and the pineal gland [26], [27]).

Many age-related changes have been described in the eye including optical or neural features that can lead to a reduction in retinal sensitivity to light [28], [29]. Aging is also associated with a reduced pupil size [30], [31] and an increased ocular lens absorption [32]–[34], both leading to a decreased retinal illumination. Lens absorption is more pronounced for short wavelength light that is optimal for appropriate entrainment of the endogenous biological clock [35]. Studies on the effects of light on the circadian system in older individuals are, however, contradictory. A decreased circadian sensitivity to white polychromatic light has been found at moderate intensities [36] but not at high or low light intensities [37]. A reduced efficiency of short wavelength monochromatic light has been reported in some studies [38] but not in others [39]. In conclusion, the effects of aging on circadian sensitivity to light remain unclear.

The aim of our study is to investigate the spectral impacts of aging on non-visual sensitivity to light and to test the hypothesis that these alterations are related to a decreased light transmittance of the aged ocular crystalline lens.

## Materials and Methods

### Melatonin suppression

#### Subjects

Thirteen healthy subjects were empanelled in a within-subject design study. Subjects were divided into two age groups: the younger group including five participants (5 men; 24–27 years old; 25.8±0.73 years, mean ± SE), and the older group including 8 participants (2 men and 6 women; 55–63 years old; 59.4±0.99 years, mean ± SE). The screening procedure included a comprehensive ophthalmologic exam to exclude subjects with significant ocular pathologies. The ophthalmologic examination was completed by visual field (Humphrey™, Carl Zeiss, Germany, Sita-standard 24-2) and color vision tests (Farnsworth-Munsell 100 Hue test). All participants were phakic in both eyes. Participants of both groups were non-smokers, non-alcoholic and free of drugs and medications known to alter the circadian system, sleep, the visual system, and melatonin secretion. All volunteers were cognitively unimpaired (Mini Mental Score (MMS)>28) and had no sleep or psychiatric disorders as revealed by the Pittsburgh Sleep Quality Index (PSQI), the Structured Interview Guide for the Hamilton Depression Rating Scale, Seasonal Affective Disorders (SIGH SAD) and the Personal Inventory for Depression and Seasonal Affective Disorders (PIDS-SA) questionnaires. None of the participants was found to be an extreme morning or evening type by the Horne-Östberg morningness-eveningness questionnaire (MEQ). Participants were asked to maintain a regular sleep wake cycle during and three weeks prior to study, and their rest/activity pattern was verified by wrist actimetry (Actitrac ™, IM Systems, Arnold, MD, USA). Subjects' sleep stability was also verified with sleep logs for the duration of the study.

Prior to participation, all subjects received a detailed description of the procedures and purpose of the experiments. Participants provided written informed consent. All procedures were in compliance with the institutional guidelines and the Declaration of Helsinki. The protocol was approved by the Institutional Review Board (Inserm RBM-022, C08-13, and Ethical Committee).

#### Schedule of the experiments

**Figure 1:**
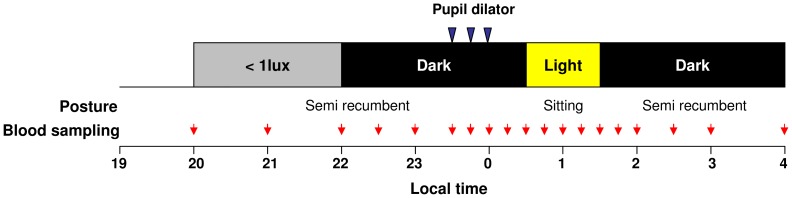
Melatonin suppression protocol. Experimental sessions started at 20:00 h and lasted until 04:00 h. Subjects were in dim light (<1 lux) between 20:00 h and 22:00 h and in total darkness afterwards. Pupils were fully dilated using antimuscarinic eye drops with one drop at three recurrences (−60, −45 and −30 minutes) prior light exposure. Subjects were exposed to 60 minutes (00:30 h–01:30 h) of monochromatic light at 9 different wavelengths (420–620 nm) of equal photon density (3.16×10^13^ photons/cm^2^/sec) over 9 experimental sessions. Outside light stimulation segment during which they were in a sitting position (90°) participants were in a semi recumbent position (45°). Blood samples were collected every 15–60 minutes and plasma melatonin was assayed by RIA. Control-adjusted melatonin suppression was compared across light treatments and sensitivity spectra were established for each subject.

Each subject underwent 10 experimental night sessions in total (1 control session and 9 light exposure sessions). During each light session, subjects were exposed for 60 minutes to one out of the 9 monochromatic stimuli (wavelengths ranging from 420 to 620 nm). The order of presentation of wavelengths was randomized for each subject across sessions. No light exposure was scheduled during the control session in order to obtain the basal melatonin secretion profile. Sessions were separated by a minimum interval of one week, to dissipate the possible phase shifting effect of light on the circadian system from one session to another. Experimental sessions were scheduled between 19:30 hours (h) and 04:00 h (Figure 1). Light intensity, posture, and fluid intake were controlled. Subjects were maintained in a dim light condition (<1 lux) between 20:00 and 22:00 h in order to measure dim light melatonin onset (DLMO) and in complete darkness (obscure room and blindfolded) from 22:00 to 00:30 h and 01:30 to 04:00 h. Outside the light stimulation period during which time they were in a sitting position (90°), participants were in semi-recumbent posture (45°) in their bed to avoid possible effects of postural changes on melatonin concentration [40]. Light exposure was scheduled between 00:30 and 01:30 h for all participants. The participants were allowed to sleep when the lights were off (before or after the light stimulation), but not to perform visual tasks requiring light (reading, watching television, etc.). Pupils of the participants were dilated using antimuscarinic eye drops (Tropicamide, 2 mg/0.4 ml, Novartis Ophthalmics, Rueil Malmaison, France), with one drop at 60, 45 and 30 minutes prior to light exposure.

Blood samples were collected via an indwelling catheter at different intervals during the entire session (every 60 minutes from 20:00 to 22:00 h and 03:00 to 04:00 h, 30 minutes from 20:00 to 23:30 h and 02:00 to 03:00 h, 15 minutes between 23:30 and 02:00 h) (Figure 1). For each sample, 4 ml of blood were collected in a heparinized plastic tube, then stored at 4°C, and centrifuged (2000 g for 20 minutes) at the end of the session. Plasma was then decanted and stored at −20°C until assayed. All experimental sessions were performed at the Chronobiology Unit (Edouard Herriot Hospital, Lyon, France).

#### Light exposure procedures

White tungsten halogen light (tungsten halogen 24V 150 Watts, Dolan-Jenne™, Boxborough, MA; correlated color temperature = 3250K) was collimated through monochromatic filters (half-bandwidth, HBWM = 10 nm, Omega Omega Optics ®, λmax 420, 440, 460, 480, 500, 530, 560, 590 and 620 nm). After diffusion by an opal glass diffuser, light was projected into a full visual field (Ganzfeld) covering sphere with a diameter of 45 cm, coated with white high reflectance paint. The sphere allowed a constant uniform illumination of the entire retina. Irradiance at subjects' eye level was monitored using a radiometer (ILT1700, International Light Technologies, Peabody, MA, USA) and the spectral emission characteristics were verified using a spectrophotometer (USB4000 Fiber Optic Spectrometer, Ocean Optics, Dunedin, FL, USA). Light was calibrated to be of equal photon density (3.16×10^13^ photons/cm^2^/sec) for each wavelength used. Pupil dilation and gaze behavior were monitored to ensure constancy of exposure for all conditions. Subject's head was maintained in a constant position inside the sphere by an ophthalmologic head holder.

#### Melatonin assay

For technical reasons, two radioimmunoassay (RIA) were used in the study. The samples of 7 of 13 subjects (5 young, 2 older) were assayed using a radioimmunological method developed in our laboratory [41]. Melatonin concentrations were determined in duplicate after diethylether extraction. Assay used an iodinated ligand. Functional sensitivity was 2.6 pg/ml. Inter-assay coefficient of variation was 11% at 50 pg/ml and 13% at 100 pg/ml (n = 15). Intra-assay coefficient of variation was 7% at 50 pg/ml and 9% at 100 pg/ml (n = 12). The samples of the remaining 6 subjects (6 older) were assayed using a commercial melatonin RIA kit (Bühlmann AG, Schönenbuch, Switzerland). With this assay, melatonin concentrations were determined in duplicate after C18 solid phase extraction by a double antibody RIA based on the Kennaway G280 anti-melatonin antibody. Functional sensitivity was 0.9 pg/ml. Inter-assay coefficient of variation was 18.1% at 1.9 pg/ml and 10.1% at 24 pg/ml (n = 18). Intra-assay coefficient of variation was 9.4% at 1.9 pg/ml and 6% at 24 pg/ml (n = 18). We verified that melatonin concentrations were similar using the two assay procedures by correlating concentrations obtained in 15 samples from one subject with the two assays (R^2^ = 0.890, p<0.0001).

### Lens density measurement

#### Subjects

Sixteen subjects participated in this study (8 young: 3 males and 5 females, 24.9±0.6 years old; 8 old: 4 males and 4 females, 61.4±0.86 years old). Subjects were screened for medical health including medical history, physical and ophthalmological exams. Subjects had normal color vision, visual field and intraocular pressure, and did not have signs of ocular diseases. The protocol was approved by the Institutional Review Board (Inserm C06-17, and Ethical Committee) and all subjects gave written informed consent. Procedures were in compliance with the institutional guidelines and the Declaration of Helsinki. All experiments were performed between 09:00 and 19:00 h.

#### Principles of the psychophysical test

Lens density of the participants was measured using a validated scotopic heterochromatic flicker photometry technique developed in our laboratory [42]. Psychophysical procedures measuring peripheral vision have been shown to provide a highly accurate measure of lens density, as the macular pigment is mainly influential in central vision [34], [43]. The principles of this approach have been described in detail in [42]. In brief, when no filtering is induced by the ocular lens, paired light stimuli positioned symmetrically on each side of rod maximum sensitivity (L1 and L2), elicit a similar scotopic thresh old (I1 = I2). When a non-homogeneous filtering occurs, as is the case with aging, a different scotopic threshold is obtained between these light stimuli (I1≠I2). A lens density index is calculated using the formula:
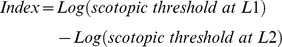
and the transmittance spectrum is derived from the ocular media model proposed by van de Kraats and van Norren [44].

#### Protocol

Subjects arrived in the dark-room 1 hour before testing, were seated before a full visual field covering box with a chin rest and a joystick, and explained the testing procedure. Full description of the apparatus is available in [42]. After a 45 minutes dark-adaptation, necessary for maximal rhodopsin regeneration and optimal scotopic sensitivity, subjects used a two-way (up/down) joystick to adjust light intensity of the flickering 410 nm light stimulus (annulus, 3° wide, 15°–18° off-center), until it fuses with the 560 nm reference light stimulus (intensity priorly determined). Green and violet LEDs were square wave-modulated in counterphase (on/off, 2 Hz flickering). Since both lights are perceived as gray in scotopic conditions, total fusion can only be achieved once both lights are detected at the same intensity. Subjects' fixation was aided by a centered small red fixation light spot (10′ diameter, 7 to 10 cd/m^2^). Subjects were given 5 trials for each eye; acquisitions lasted 10 minutes after dark adaptation.

### Data and statistical analyses

Individual dim light melatonin onset (DLMO) and amplitude were calculated respectively as the 25% upward crossing and maximal secretion of the smoothed melatonin profiles (locally weighted smoothing LOESS method, span = 0.5). The 25% threshold was calculated for each subject from his/her profile obtained during the dark condition of the study (between 20:00–04:00). DLMOs were compared within subjects across session and between subjects using a two-way ANOVA for repeated measures (time, subject). DLMO were compared between young and aged groups using a two-way ANOVA for repeated measures (time, age groups).

**Figure 2:**
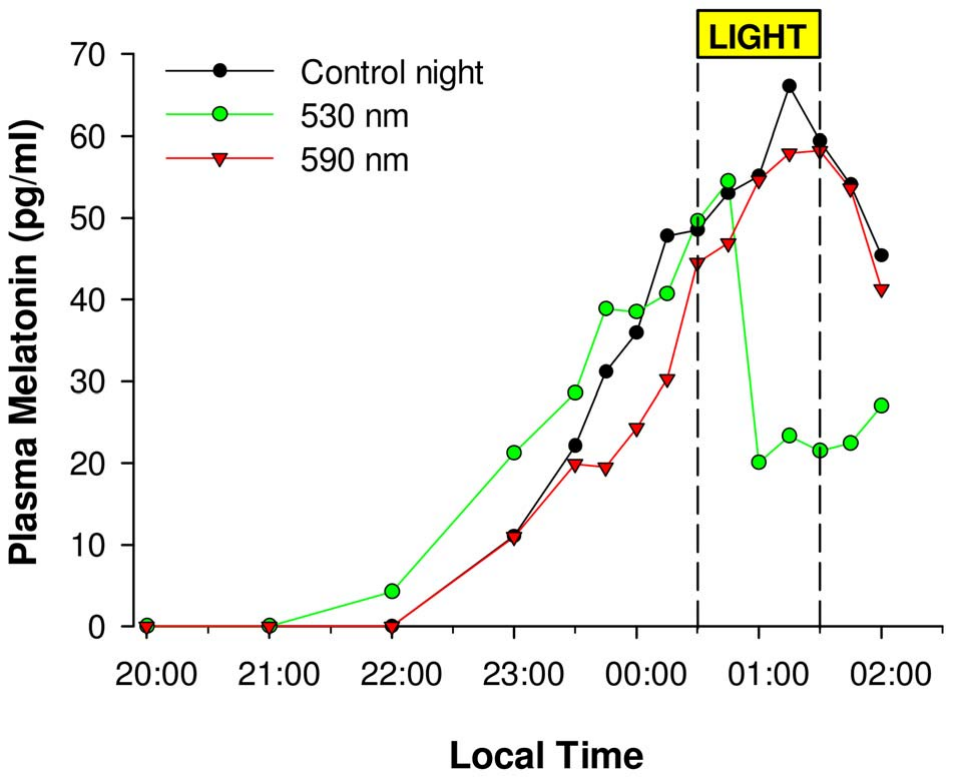
Evaluation of non-visual sensitivity to light *via* melatonin suppression. Profiles of plasma melatonin in a representative older subject during 3 experimental sessions, with exposures to 2 monochromatic lights (530 (green line) and 590 nm (red line)) of equal photon density, and a control dark session. Note the wavelength-dependent acute effects of the monochromatic lights compared to control (black line). A relatively stable phase angle of entrainment is observed for this subject across this 4 weeks' study segment.

Melatonin suppression scores were determined by the following formula:
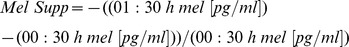
Melatonin suppression values were then normalized to control-adjusted (CA) suppression scores by subtracting the control (no light) condition percent suppression scores for each subject from that same subject's light exposure score (Figure 2):
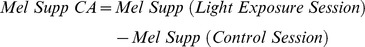
This technique accounts for the normal individual rise or fall in plasma melatonin levels with respect to the light-induced changes [45], [46]. Melatonin suppression (CA) was compared across wavelength using a one-way ANOVA for repeated measures with Greenhouse-Geisser correction (only original degrees of freedom are reported), with wavelength as a repeated factor. Log (base 10) of individual suppression control adjusted values were then fitted to obtain individual melatonin suppression action spectra using a four parameter Gaussian procedure:
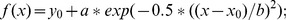

*where y_0_: minimum melatonin suppression, a: maximum amplitude, b: standard deviation and x_0_: peak wavelength*

The predicted impact of lens density on melatonin suppression was derived using a non-linear approach extracting melatonin attenuation from the fluence response curves by Brainard et al. 2001 (see detailed procedure in File S1).

Lens density results and melatonin secretion amplitude were compared between young and old groups using a Welch Two Sample t-test. Area under the transmittance curves (AUC) of young and old subjects was compared using a Student t-test.

Peak sensitivity, wavelength at 50% suppression on upward and downward slopes as derived from fitted spectrum in young and aged subjects were compared using the median test.

Statistical analyses were performed using Statistica (StatSoft, Inc., Tulsa, OK, USA). Data are expressed in mean ± SE.

## Results

### Melatonin secretion and suppression

All young and older subjects displayed significant nocturnal melatonin secretion. Melatonin amplitude during the control session was not significantly different between young (65.8±20.9 pg/ml) and older subjects (89.3±27.3 pg/ml) (p = 0.5). DLMO were not significantly different between age groups (F (1, 11) = 1.36, p = 0.26) and did not vary significantly across sessions (F (9, 99) = 0.43, p = 0.92). Mean circadian phase, as estimated from DLMO, was 22:01±00:27 h in the young and 22:28±00:28 h in the older participants.

### Non-visual sensitivity in young and older subjects

Acute melatonin suppression in both age groups was wavelength-dependent (Young: F (8, 32) = 5.87, p = 0.00012; Older: F (8, 56) = 12.6, p<0.0001). Average control adjusted melatonin suppression was significantly fitted using a four parameter Gaussian in the young (p = 0.0011, R^2^ = 0.94 on raw values; p<0.05 and R^2^ = 0.81 on log-transformed values), and older (p = 0.0069, R^2^ = 0.90 on raw values; p<0.01 and R^2^ = 0.89 on log-transformed values).

**Figure 3:**
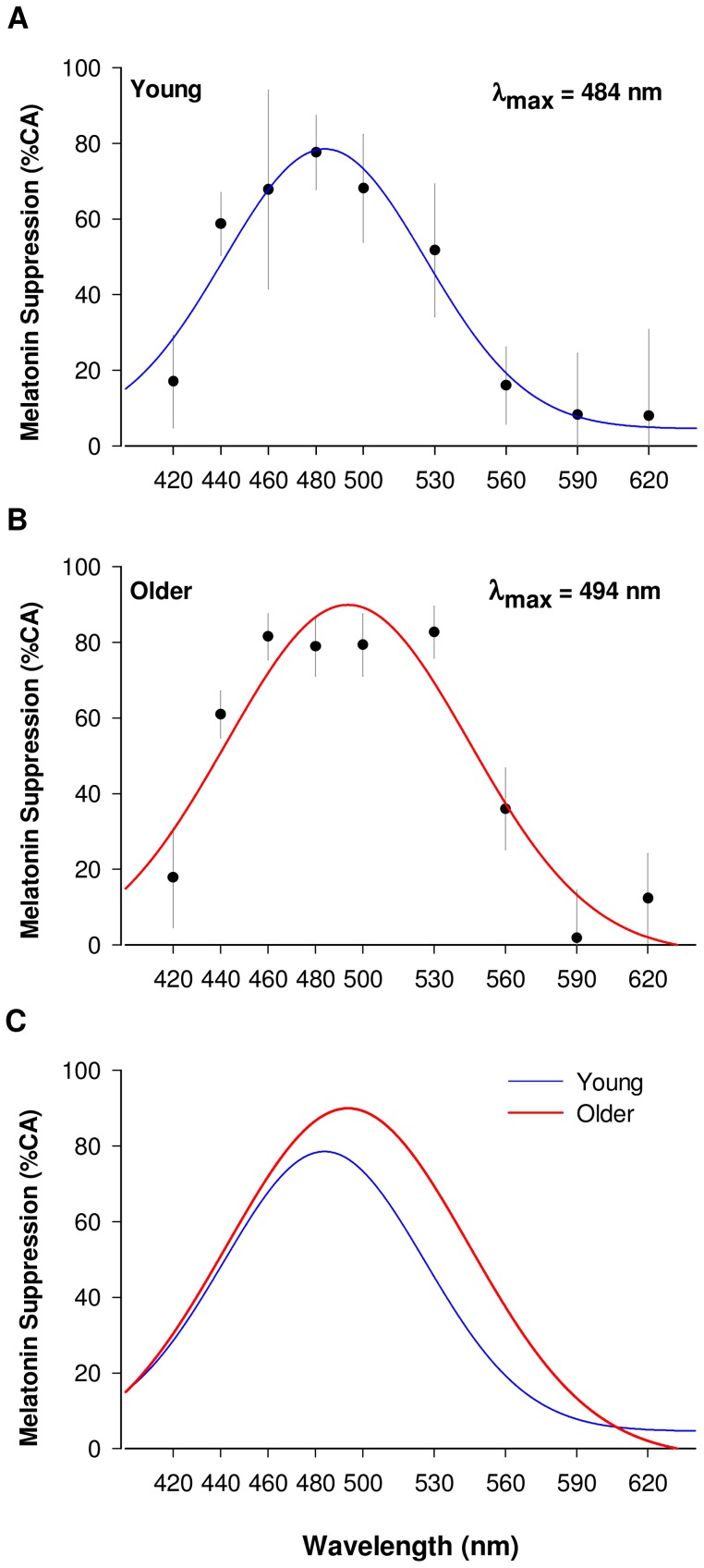
Non-visual spectral sensitivity to light (melatonin suppression). **A.** Melatonin suppression in young (n = 5) and (**B**) older participants (n = 8) is represented in percentage control adjusted (%CA). In both young and older groups short (420 nm) and long wavelengths lights (>560 nm) have a limited effect on melatonin suppression. Using a four parameter Gaussian fitting procedure, peak sensitivity is found at 484 nm in the young (blue fit curve, R^2^ = 0.94 and p<0.01 on raw values; R^2^ = 0.81 and p<0.05 on log-transformed values)and 494 nm in the older participant (red fit curve, R^2^ = 0.90 and p<0.01 on raw values; R^2^ = 0.89 and p<0.01 on log-transformed values). **C.** Comparison of spectral sensitivity in young (blue fit curve) and older subjects (red fit curve). Older subjects show an altered spectral sensitivity of melatonin suppression compared to young subjects. Sensitivity to light in the older is similar in the short wavelength region of the spectrum (<500 nm), but higher for 500–590 nm, and with a shift of peak sensitivity from 484 nm (young) to 494 nm (older).

Peak sensitivity calculated from this fit was 484 nm in young (Figure 3. A) and 494 nm in older subjects (Figure 3. B). Amplitude of suppression at peak wavelength was not significantly different between the two groups. Monochromatic lights at short and long wavelengths (420, 590 and 620 nm) had a minor effect on melatonin secretion (0.2 to 18%) in both age groups (Figure 3).

### Comparison of non-visual sensitivity to light between young and older subjects

Spectral sensitivity to light was altered in the older subjects compared to young. Peak melatonin suppression was significantly shifted from 484 nm in the young to 494 nm in the old (p = 0.008) and a small but not statistically significant, increase in melatonin suppression was observed in the long wavelength 530 to 560 nm range (p = 0.13). Sensitivity to light was similar between young and aged subjects in the short wavelength region of the spectrum (<500 nm) (p = 0.72) (Figure 3. C).

### Lens density changes with aging

**Figure 4:**
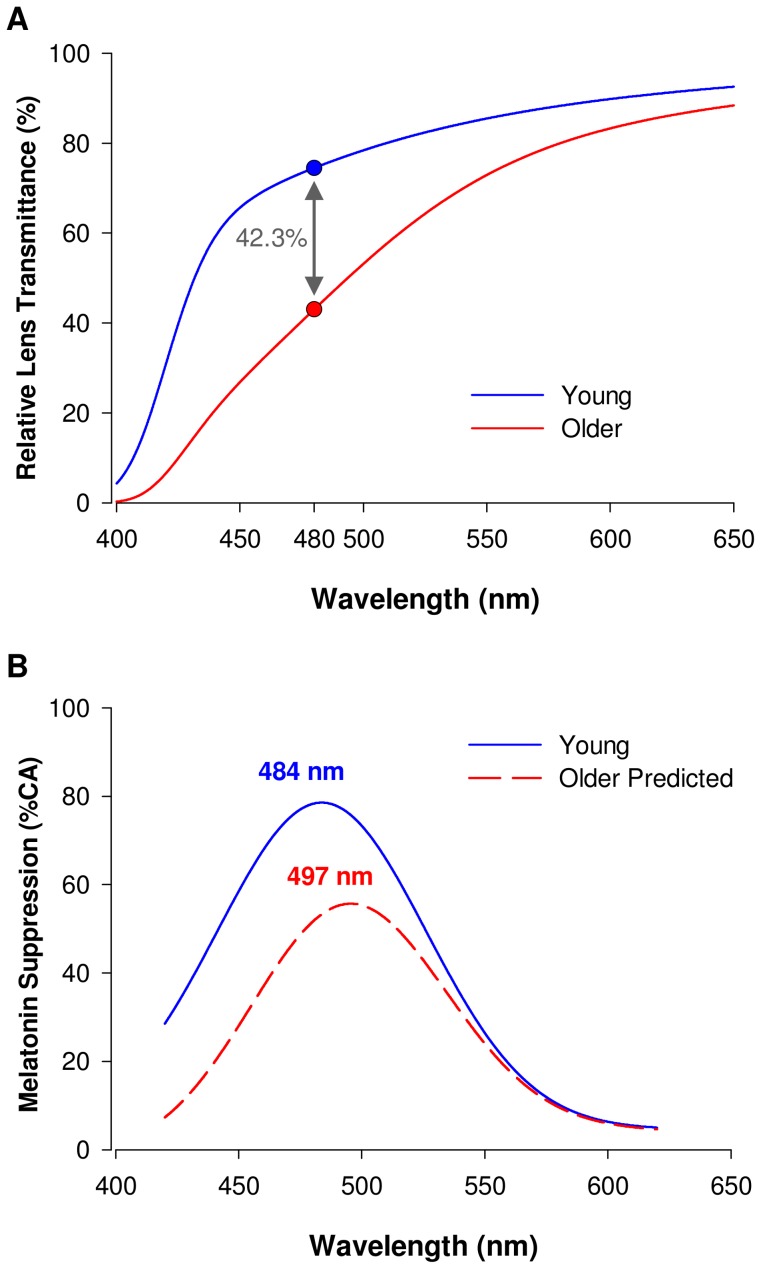
Changes in lens transmittance with aging and predicted impact on non-visual spectral sensitivity to light. **A.** Transmittance spectra of the ocular lens in the young (n = 8, red line), and older subjects (n = 8, blue line). Filtering is particularly pronounced in the short to medium wavelength range (<530 nm) in the old compared to young subjects. Symbols on the curves indicate the average lens transmittance at 480 nm in the young (blue circle, 74.4%) and in the old subjects (red circle, 42.9%). An average decreased transmittance by 42.3% is found between young and old. **B.** real melatonin suppression spectrum in young subjects (blue line), and predicted melatonin suppression spectrum in the older subjects based on their decreased lens transmittance (red dashed line). Note the expected decreased melatonin suppression to short wavelength lights in the elderly and the shift in peak sensitivity to light from 484 nm to 497 nm. A 34% attenuation in melatonin suppression is predicted at 480 nm (see Figure S2 and File S1 for details).

Lens density was significantly increased in older participants compared to young (p<0.0001) (Figure S1). This increase in lens density in the older participants was associated with a decrease in transmittance of the crystalline lens over the entire visible spectrum. Overall AUC between 400 and 650 nm was attenuated by 16.2% between the young and older group (AUC young = 237.3±1.4 percent transmittance; AUC older 198.9±3.3 percent transmittance; p<0.0001) (Figure 4. A). The decrease in transmittance was more pronounced in the short wavelength range (400–500 nm) (AUC attenuated by 53.4% between the young and older group; AUC young = 41.9±0.9 percent transmittance; AUC older = 19.5±1.6 percent transmittance; p<0.0001) compared to the longer wavelengths (500–620 nm) (AUC attenuated by 14.8% between the young and older group; AUC young = 79.4±0.3 percent transmittance; AUC older = 67.7±1.2 percent transmittance; p<0.0001). An average 42.3% decrease in transmittance between young and older participants was found at 480 nm light (p<0.0001) (Figure 4. A). This reduction in transmittance was estimated to reduce retinal illumination by 45% over the melanopsin sensitivity spectrum.

### Effects of lens density on non-visual sensitivity to light

Since melatonin suppression is irradiance dependent, we made the assumption that the decrease in lens transmittance to light with aging would lead to a diminished melatonin suppression. Taking into account the decreased lens transmittance measured in our subjects (Figure 4. A), we predicted that melatonin suppression would be altered in the aged as illustrated in Figure 4 B. This prediction curve shows a decreased melatonin suppression, especially in the short wavelength range of the spectrum (<500 nm) and a shift in peak sensitivity from 484 to 497 nm in the aged subjects (Figure 4. B). This prediction, however, did not hold as our results showed no decrease in non-visual sensitivity to short wavelength light (Figure 3. C).

### Lens transmittance and melatonin suppression at peak melanopsin sensitivity

**Figure 5:**
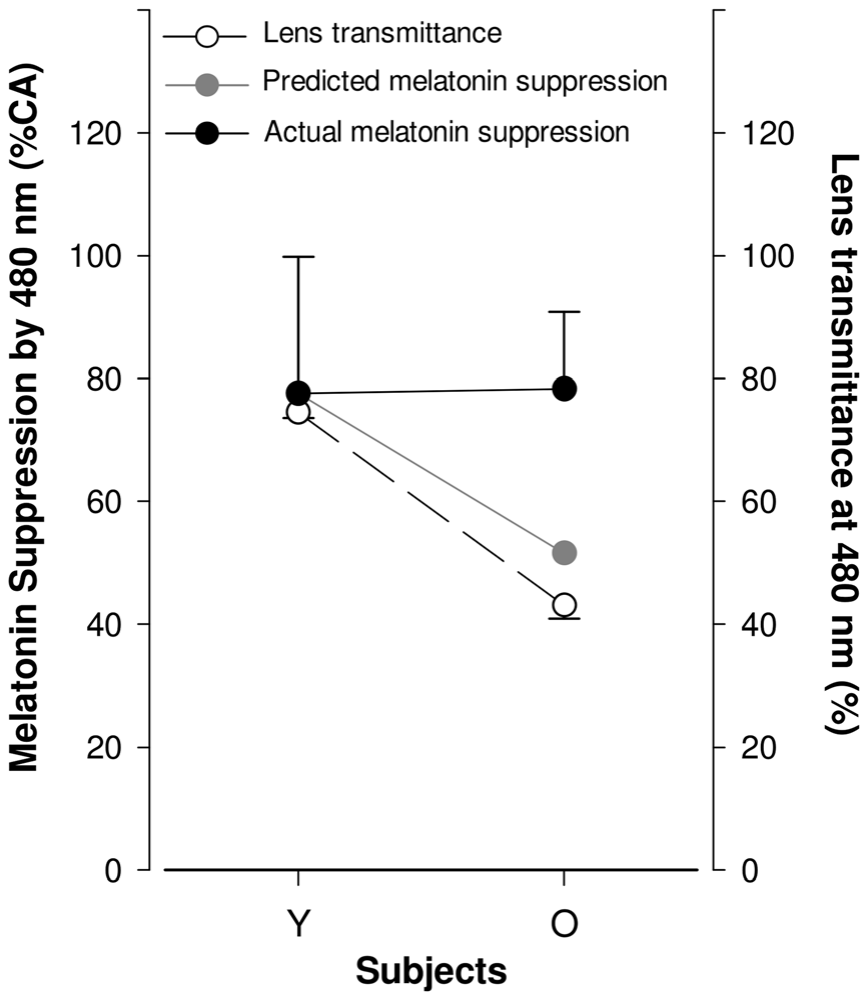
Melatonin suppression and lens transmittance at 480 nm light, in young and aged subjects. Lens transmittance (open circle, straight line) is significantly lower in older compared to young subjects (p<0.0001) whereas melatonin suppression (full circle, straight line) at 480 nm is not significantly different between aged and young. The predicted effect of a decrease in lens transmittance at 480 nm on melatonin suppression is shown in gray circle, dashed line. Prediction was made using Brainard et al. 2001 irradiance response curves. See File S1 for more details on the analytics of the prediction.

As illustrated in Figure 5, transmittance at 480 nm was significantly decreased by 42.3% in the aged subjects (p<0.0001). Such decreased transmittance would predictably lead to a 34% decrease in melatonin suppression. Our results, however, did not show such a reduction in melatonin suppression at 480 nm (Figure 5).

## Discussion

The present study shows a decreased lens transmittance in the older subject, primarily in the short wavelength region of the spectrum (400–480 nm). This result confirms previous *in vivo* and *in vitro* findings in humans showing a decreased lens transmittance with age [32]–[34], [44], [47], [48]. Our results also reveal that lens density significantly increases between the second and sixth decades of life, ultimately leading to a 45% decrease in light transmission over the melanopsin sensitivity spectrum and a 42.3% decrease in transmittance at peak melanopsin sensitivity (480 nm). Nevertheless, non-visual sensitivity to light is not reduced in this same wavelength range. Overall, our study reveals that the increased lens filtering occurring with aging does not lead to a proportional decrease in non-visual sensitivity to light.

This study precisely assessed non-visual sensitivity to light over the entire visible spectrum in young older individuals. We expected that the observed decrease in lens transmittance to short wavelength light would lead to a decreased non-visual sensitivity to light in the aged. While our results show that non-visual sensitivity to light is marked by a significant shift of peak sensitivity in the elderly compared to the young (λmax = 484 nm in the young, λmax = 494 nm in the older participants), melatonin suppression in response to short wavelength light (<500 nm) unexpectedly remained similar in both age groups.

Previous studies, however, have reported a decreased non-visual sensitivity to light in healthy older subjects. Herljevic and colleagues compared the effects of 30 minutes exposure to 456 and 548 nm monochromatic lights in pre- and post-menopausal women [38]. The authors found a decreased melatonin suppression to 456 nm, but not to 548 nm light in the aged subjects. The discrepancy between these results and ours could be due to differences in experimental procedures, in particular in the duration and photon density of light exposures used. Moreover, other studies have used a published template to estimate subjects lens yellowing [49] but did not measure individual subjects lens densities The template in question is a two-factor non-linear lens density spectrum model that uses age as a variable and provides a relatively good estimate of average lens density for a group of subjects but does not provide an accurate estimate of lens density for any given individual. Indeed, our data demonstrate that age is not always an accurate predictor of lens density. In fact, inter-individual variability is increased in the elderly (0.4 log units span), and two subjects of the same age could have significantly different lens density (see Figure S1).

In a more recent study, the same group [39] tested the effect of blue (456 nm) and green (548 nm) lights on phase shift, alertness, mood and sleepiness. The authors found that responses in older men were significantly diminished compared to young men for subjective alertness, sleepiness and mood during and after blue (456 nm) light exposure. The effects of blue monochromatic light on circadian phase were, however, not different between younger and older subjects. This latter result is in agreement with our findings and supports the idea that phase shifting and melatonin suppressing effects of short wavelength light are not necessarily altered in healthy older individuals.

Benloucif et al. [37] showed that older adults were able to phase delay to the same extent as younger subjects when exposed to 4 h of control dim (10 lux) or bright polychromatic light (3500 lux) during the night. These results are consistent with those from Duffy and colleagues [36] showing no alteration in the response to very dim or bright light in older adults, although a decreased phase shifting effect was found in the aged at moderate light intensities (100–1000 lux). Moreover, Duffy et al. [36] found no significant relationship between melatonin suppression and illuminance, nor between melatonin suppression and phase shift, and suggested that distinct pathways from the retina to the SCN could be involved in acute melatonin suppression and entrainment. Although we are not aware of any evidence in the literature that two distinct pathways exist, such a distinctive anatomical wiring could explain this result if selectively affected by aging. It could also alternatively be explained by different sensitivity levels of melatonin suppression and phase shifting in response to light, as discussed in a study by Zeitzer et al. [10].

The possibility remains that non-visual responses to light undergo adaptive or compensatory mechanisms during healthy aging. The retina has been shown to be plastic with age. In fact, with aging various cells undergo dendritic sprouting in different retinal layers, in humans [50] and mice [51]. Such reorganization is also found in the peripheral retina of human patients with retinitis pigmentosa [52] and could be induced by injury at the retinal level [53]. To date the function of this retinal plasticity with aging remains unclear but could serve as a compensative mechanism induced by photoreceptor loss.

Another possible compensatory mechanism involves the marked regulation of melanopsin mRNA and protein expression by environmental illumination [54]–[56]. Prolonged (5 days) darkness causes a robust increase in melanopsin mRNA and protein levels in mice, whereas constant light markedly decreased melanopsin mRNA level and melanopsin immunoreactivity also in mice. Such a mechanism of up-regulation in melanopsin, if occurring after progressive reduction in light reaching the retina due to lens yellowing, could possibly compensate for a decreased sensitivity of the retina. Moreover, classical photoreceptors (rods and cones) can regulate the expression of melanopsin mRNA [7], [57]; and therefore, the structural reorganization in the aging retina illustrated by a phenomenon known as dendritic sprouting [50], [51] could modulate the incoming signal from rods and cones to ipRGCs.

Circadian sensitivity to light has been shown to be affected by recent changes in light history, both in field studies and controlled laboratory conditions [58]–[60]. Also, a lower exposure to daily light in winter followed by dark adaptation (sleep of 3 h) has been reported to increase subsequent melatonin suppression [61]. Therefore, as lens yellowing is associated with a diminished light transmittance, the progressive decreased ocular transmittance occurring with aging could constitute a long-term alteration of light history that could in turn lead to an increased light sensitivity, as a physiological adaptation. The physiological processes involved in this phenomenon of light history remain to be clarified, but they could involve some of the mechanisms aforementioned.

Finally, there is evidence suggesting that mammalian melanopsin functions like an invertebrate bistable photopigment. In such photopigments, regeneration to a photoresponding state (from the *all-tran*s to the light sensitive *11-cis* bound state) is an active light-driven process [62]–[64]. For melanopsin, this photoisomerization is driven by long wavelength red light [63], [65], [66]. With this mechanism in mind, lens filtration of short wavelength light in older individuals could favour an increase in the pool of the light sensitive 11-cis form of melanopsin, subsequently increasing light sensitivity. Bistability could therefore be involved in compensatory mechanisms occurring in the aging of non-visual sensitivity to light.

Aging is associated with various molecular, cellular and neuronal changes. Such changes may also contribute to sleep and circadian disruption, and are likely to affect the precision and robustness of rhythmic information transmission by the SCN to other neural sites [25]. Such changes offer alternative mechanisms to our hypothesis of the decreased lens transmittance leading to a decreased non-visual response to light. Davidson and colleagues showed a reduced expression of Bmal1 and Clock in the SCN during the subjective night and daytime respectively, in older hamsters compared to young [67]. Moreover, Per2 expression is reduced in the pituitary gland of older rhesus macaques compared to younger animals [68] and a reduction in peripheral expression of Per 1,2,3 in the liver and heart of older rodents, suggest a reduction in peripheral clock gene expression with aging [69]. Age-related circadian changes may also be linked to a reduction in the amplitude of circadian rhythms in SCN electrical activity [25]. AVP (arginine vasopressine neurons in the dorsomedial region of the SCN) and VIP (vasoactive intestinal polypeptide, in the ventrolatreal region of SNC), constitute the main output pathway of the SCN to the rest of the brain. Several animal and human studies reported an age-related shift [70] and decrease in the rhythmic synthesis, oscillation and release of these neuropeptides [25], [71] along with a decrease in the number of AVP-expressing neurons in the SCN [72]. Post-mortem analyzed of human brains, also show a flattened diurnal oscillation of AVP expression in older subjects compared to young [73]. VIP expression in the SCN have been reported to play an major role in the transmission of photic information to the circadian timing system [71], [74]. Thus age-related AVP and VIP changes could be accountable for the decreased non-visual photic impact in the older population. Studies in animal models have also shown age-related reduction in pituitary adenylate cyclase-activating peptide (PACAP) in the SCN [75]. Interestingly, photic signal transduction reaching SCN passes through the release of glutamate and PACAP. Because PACAP seems to be exclusively co-expressed with melanopsin in retinal ganglion cells, age-related PACAP modifications may be associated to changes in the impact of light on circadian regulation as well as in other non-visual responses to light [76]. Altogether, these results show that aging affect multiple physiological compartments (eye, SCN firing, clock genes), that could all play a role in the sleep or circadian disorders occurring with aging.

This study sheds light on the spectral sensitivity function for melatonin suppression in the aged and demonstrates that a decreased lens transmittance is not associated with a decrease in non-visual sensitivity. Our study, however, has limitations that should be mentioned in order to put the results into perspective. First, we have included a relatively small number of subjects in both age groups (n = 5 young, and n = 8 older). Although this would lead to a limited statistical power in protocols where single comparisons would be made between two age groups, our repeated-measure within-subject design, and our mathematical modeling of non-visual sensitivity to light (fitting procedure), provide more statistical power and therefore more confidence in that the light spectra we obtained in the young and the older have the differences we described. Second, the most accurate method to derive a spectral sensitivity curve is via measurement of irradiance response curves obtained at multiple wavelengths. This technique, known as the fluence response curves approach, has been used by Brainard and Thapan in the first studies in humans showing a peak sensitivity for melatonin suppression at around 465 nm [13], [15]. We employed a slightly different approach in our study, using light stimuli at the same photon density for all nine wavelengths. Although this is a correct way to control for different light irradiances, a given photon density could be optimal for one wavelength and saturating for another, and thus lead to a biased spectral sensitivity curve. Although we believe that the light intensity we used does not saturate the non-visual response based on previous irradiance response curves [13], we cannot exclude that the use of relatively high light intensities could reduce a possible difference between young and old. To verify this point, we exposed our young and older participants to a one-hour 480 nm light stimulus that contained a third of the photon flux used in the current study (i.e., 1×10^13^ photons/cm^2^/s versus 3.16×10^13^ photons/cm^2^/s). For both light intensities, we found no difference in melatonin suppression between groups suggesting that indeed the lack of difference in melatonin suppression between young and old in this study is not due to response saturation nor light intensity *per se*. Another piece of evidence that our approach yields a correct spectral curve is the finding of a peak sensitivity around 480 nm in the young, comparable to that found in studies in the young [13], [15], [77], and in agreement with the peak sensitivity of melanopsin in human [78] and non-human primates [79]. Third, for technical reasons, the young subjects who underwent the melatonin suppression study were not the ones who underwent the lens density measurement, six out of eight older subjects underwent both studies (melatonin suppression and lens density), two older were measured only for melatonin suppression, and two older subjects only for lens density. We do not believe, however, that this may have biased our results and conclusions. Indeed, we know from ophthalmologic examination that all the healthy young participants recruited in our 2 studies had clear lenses. We also know from the current sample (see Figure S1) and from recent results obtained on a larger population (n = 43 from which 17 young; Najjar et al. unpublished data), that inter-subject variability in lens density is very small in young subjects. Therefore, we expect that the group of young subjects in which we have measured melatonin suppression would have had a similar lens density to the group of young subjects in which we measured it. The older subjects who were measured only for melatonin (n = 2) or lens density (n = 2) did not respond differently from the other older subjects (responses within the range of the other 6 subjects).This was expected, as not only they were the same age and had the same health status (verified by a clinical exam), but they also underwent a stringent ophthalmological examination that confirmed that they had no ocular pathologies, no cataract, a normal visual field and acuity. Fourth, we excluded subjects with cataract from our study, and therefore our aged subjects might not be considered as representative of the general elderly population. This exclusion criterion might explain the absence of a difference in non-visual sensitivity to light between old and young subjects. Although we do not exclude that aged subjects with sleep and circadian alterations, or with cataract, might have a decreased non-visual sensitivity to light, there is no clinical evidence that healthy older individuals have a decreased non-visual sensitivity to light. Indeed, it has been reported that only half of cataract patients suffer from poor sleep, and that increased light transmission after cataract surgery [47] improves sleep quality [80] and decreases psychomotor reaction time [81]. On the other hand, patients with normal sleep quality show no difference in their sleep following cataract surgery [80], nor in their melatonin secretion, sleep parameters or sleepiness [82]. Therefore, healthy aging does not necessarily lead to altered sleep and circadian physiology.

## Conclusion

The present study clarifies the effect of aging on spectral sensitivity to light of the circadian system. This could lead to important optimization in the spectra of light therapy approaches utilized in the older individual (e.g. elderly homes). Our results also suggest that increased light filtration through the ocular lens that occurs during healthy aging, does not lead to a decrease in non-visual sensitivity (melatonin suppression) to these wavelengths. We believe that during healthy aging, compensatory or adaptation mechanisms, similar to those described in the visual system (e.g. constancy of the achromatic locus across the life span or compensation for light loss due to filtering by macular pigment) [83], [84], take place to maintain an optimal non-visual sensitivity to light. Although visual compensation is thought to occur at a cortical level, non-visual compensation could occur at the eye, the SCN, or at the level of clock-output structures. Further investigations are needed to clarify this aspect.

## Supporting Information

Figure S1 Raw data of lens density increase with aging. Individual (open circles) and average lens density measures in young (blue circle) and older (red circle) subjects. Lens density is significantly increased in the aged compared to young (p<0.0001). Note that variability is higher in the elderly compared to the young participants.
(TIF)

Figure S2 Predicted spectral attenuation of melatonin suppression.
(TIF)

File S1 Analytical procedure to predict the melatonin suppression spectrum in the older.
(DOC)
