# Supplementary figures and images for: Aging of Non-Visual Spectral Sensitivity to Light in Humans: Compensatory Mechanisms?
Source: PLoS One. 2014 Jan 23;9(1):e85837. doi: 10.1371/journal.pone.0085837 (PMC3900444; doi:10.1371/journal.pone.0085837)

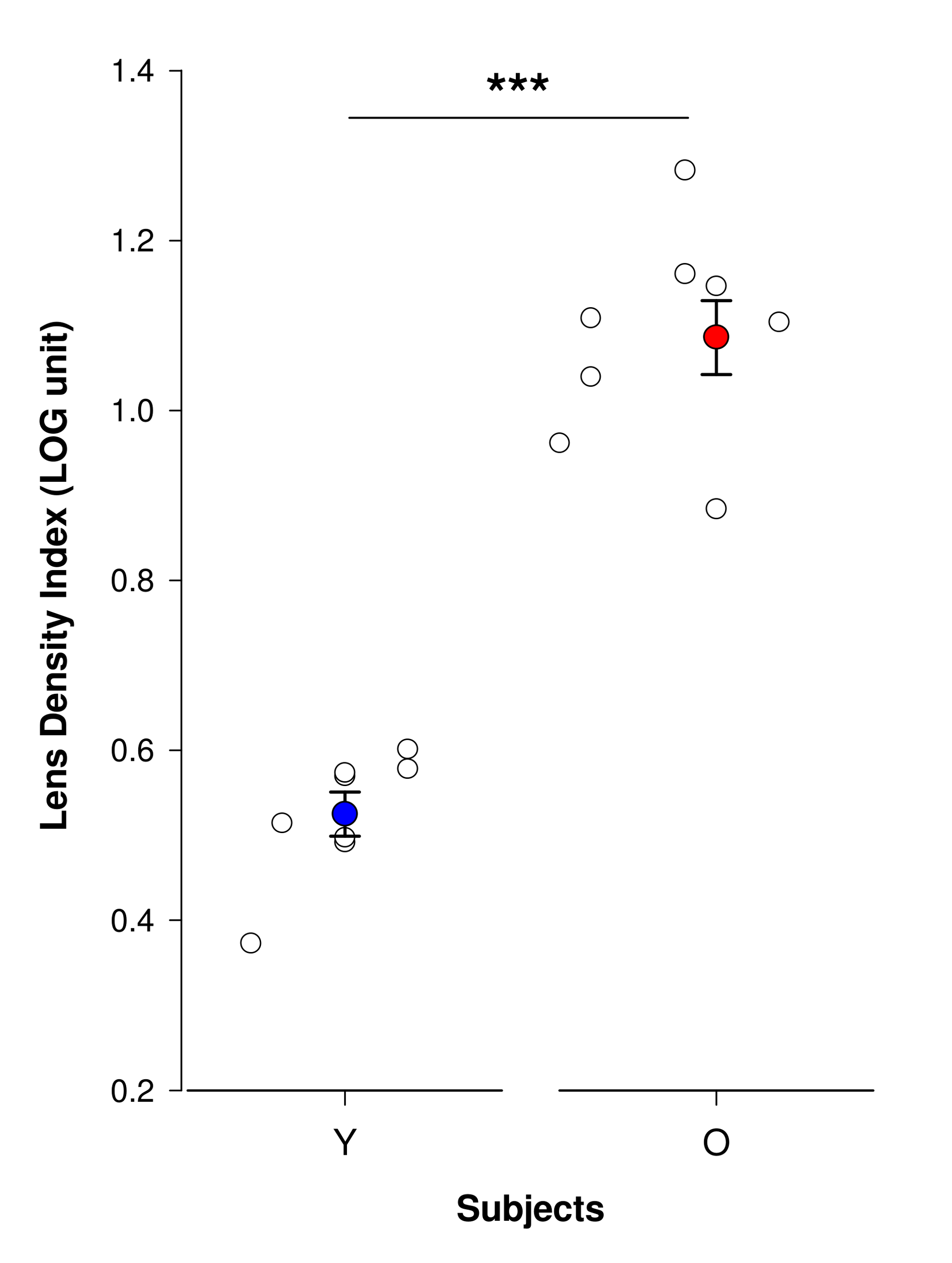

Supplement: Figure S1 — Raw data of lens density increase with aging. Individual (open circles) and average lens density measures in young (blue circle) and older (red circle) subjects. Lens density is significantly increased in the aged compared to young (p<0.0001). Note that variability is higher in the elderly compared to the young participants. (TIF) [file pone.0085837.s001.tif]

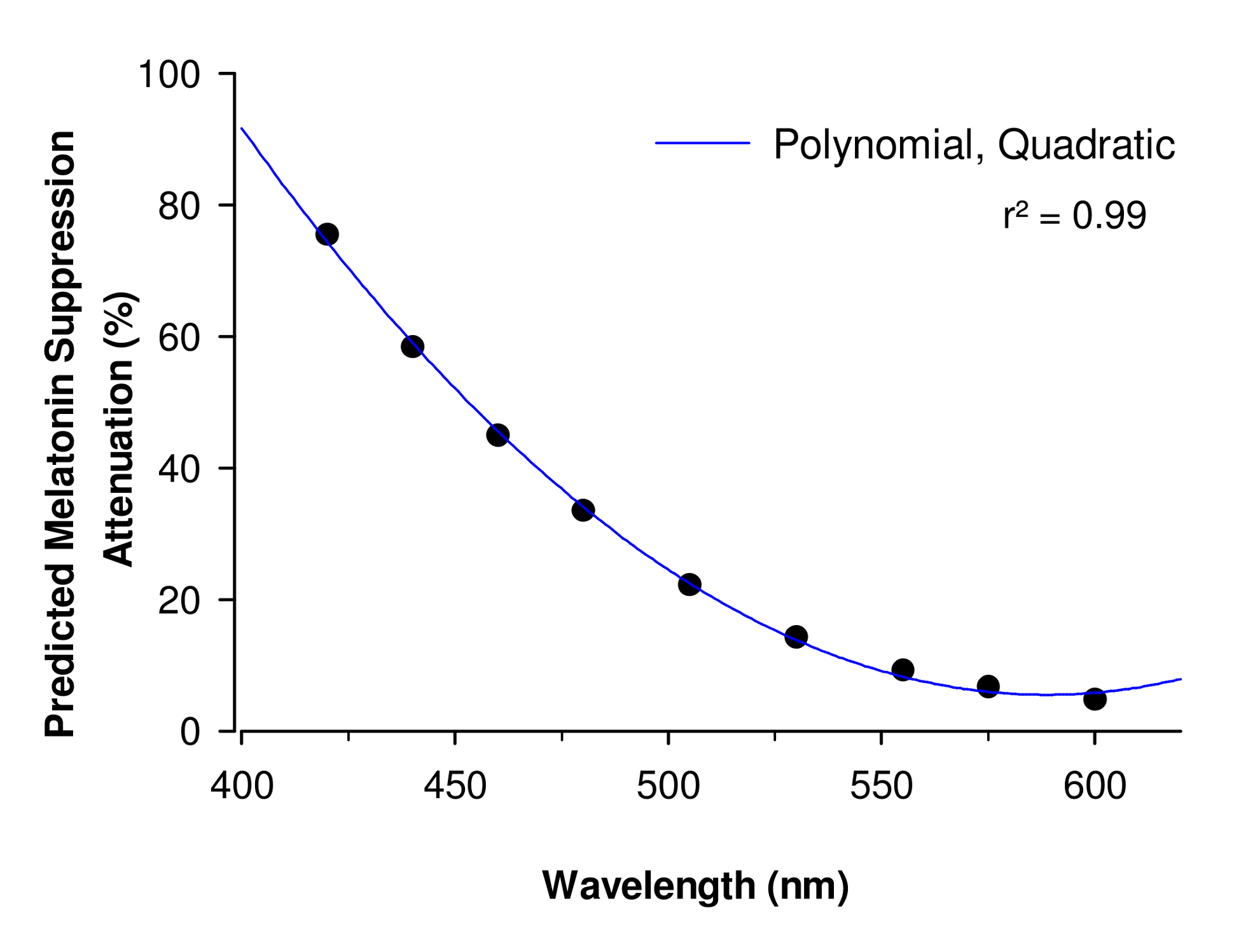

Supplement: Figure S2 — Predicted spectral attenuation of melatonin suppression. (TIF) [file pone.0085837.s002.tif]
