# Supplementary material for: Aging of Non-Visual Spectral Sensitivity to Light in Humans: Compensatory Mechanisms?
Source: PLoS One. 2014 Jan 23;9(1):e85837. doi: 10.1371/journal.pone.0085837 (PMC3900444; doi:10.1371/journal.pone.0085837)
Supplement: File S1 — Analytical procedure to predict the melatonin suppression spectrum in the older. (DOC) [file pone.0085837.s003.doc]

**Aging of non-visual spectral sensitivity to light in humans:
compensatory mechanisms?**

Raymond P. Najjar ^1, 2, a^, Christophe Chiquet ^1, 3, 4^, Petteri Teikari ^1, 2, b^, Pierre-Loïc Cornut ^1, 5^, Bruno Claustrat ^1, 6^, Philippe Denis ^1, 7^, Howard M. Cooper ^1, 2, #^ and Claude Gronfier ^a, b, #, *^

^1^ Inserm U846, Stem Cell and Brain Research Institute, 69675 Bron, France;

^2^ University of Lyon, Claude Bernard Lyon 1, 69622 Villeurbanne Cedex, France;

^3^ University Joseph Fourier Grenoble 1, F-38041 Grenoble, France;

^4^ Department of Ophthalmology, CHU Grenoble, F-38043 Grenoble, France;

^5^ Department of Ophthalmology, CHU de Lyon Hôpital Edouard Herriot, 69003 Lyon, France;

^6^ Center of Biology, Hormone Laboratory, 59 Boulevard Pinel, 69677 Bron, France;

^7^ Department of Ophtalmology, Hôpital de la Croix-Rousse, 69317 Lyon Cedex 04, France

**Analytical procedure to predict the melatonin suppression spectrum in the older**

The procedure we followed to calculate the predicted impact of lens transmittance on melatonin suppression is described below:

1. We re-plotted Brainard et al. 2001 fluence response curves, extracted their constant half saturation responses X50 at each wavelength, to which we applied the lens-related attenuation of the older individual:

*X50att (lambda) =X50(lambda) x lens transmittance (lambda)*

1. We assessed the melatonin suppression that would be caused at X50(att) from Brainard’s IRC, and calculated the predicted percentage of attenuation in melatonin suppression at each wavelength (Figure S2):

*%Mel (X50att (lambda)) = [Mel(X50(lambda))-Mel (X50att (lambda)))/Mel(X50(lambda))]*

1. Then, because we did not use the exact same wavelengths, for melatonin suppression, as Brainard et al., we had to resample the predicted attenuation in melatonin attenuation in order to extract the attenuation at our wavelengths. To do so, we interpolated the data using a quadratic polynomial template:

*% Melatonin suppression attenuation = f (lambda) (*Figure S2)

1. Finally we used the fitted attenuation in melatonin suppression on our young subject’s action spectrum to derive the predicted older melatonin suppression spectrum (Figure 4).

Note that due to protocol differences, mainly in light exposure duration between our study (60 minutes) and Brainard et al. 2001’s study (90 minutes), it is possible that this procedure slightly underestimates the effect of lens density on melatonin attenuation. Nevertheless, this would not change our conclusion (but rather reinforce it), that the spectrum of melatonin suppression that we obtained in the older is not predicted by lens density.
